# The potential of antibody-drug conjugates in immunotherapy for non-small cell lung cancer: current progress and future

**DOI:** 10.3389/fonc.2025.1630056

**Published:** 2025-09-15

**Authors:** Hongyu Lin, Xinyu Ma, Xinhai Zhu, Linru Zhong

**Affiliations:** ^1^ The Second School of Clinical Medicine, Zhejiang Chinese Medical University, Hangzhou, China; ^2^ Department of Thoracic Surgery, Zhejiang Hospital, Hangzhou, China

**Keywords:** review, NSCLC, immunotherapy, cancer, antibody-drug conjugates

## Abstract

Antibody-drug conjugates (ADCs) have gained significant attention as a promising therapeutic strategy for non-small cell lung cancer (NSCLC), combining the precision of monoclonal antibodies with the potent cytotoxic effects of chemotherapy. This review summarizes recent advancements in the development of ADCs for NSCLC, focusing on their mechanism of action, key components, and progress in clinical applications. By specifically targeting tumor-associated antigens, ADCs deliver cytotoxic agents directly to cancer cells, thereby enhancing therapeutic efficacy while minimizing systemic toxicity. Several ADCs, such as trastuzumab deruxtecan and sacituzumab govitecan, have shown encouraging results in clinical trials, particularly in tumors with molecular alterations like HER2 and TROP2. Additionally, the combination of ADCs with immune checkpoint inhibitors (ICIs) offers a novel and promising therapeutic avenue, potentially enhancing immune responses and overcoming tumor resistance. Despite these promising outcomes, challenges such as drug resistance, immune evasion, and toxicity persist. The novelty and focus of this article are to discuss the significance of optimizing ADCs design, exploring combination therapies, and enhancing safety management in improving treatment outcomes, with the aim of promoting the research and application of ADCs in the immunotherapy of NSCLC

## 1 Introduction

Cancer remains a major global public health issue (1). Among the various types, lung cancer is the leading malignancy in terms of both incidence and mortality rates globally, with approximately 2.5 million new cases of pathology each year (2). Non-small cell lung cancer (NSCLC) is the most common histological subtype of lung cancer, accounting for approximately 85% of the lung cancer cases observed globally at present (3). This emphasizes the urgency of effective measures for treating NSCLC.

Treatment options for NSCLC include surgery, chemotherapy, radiotherapy, immunotherapy, targeted therapies, antibody-drug conjugates (ADCs), and traditional Chinese medicine. Immunotherapy, especially immune checkpoint inhibitors (ICIs), has significantly enhanced the treatment outcomes for NSCLC (4). However, the emergence of resistance to these therapies continues to pose a major challenge (5). ADCs is a highly promising treatment for NSCLC. ADCs are immunoconjugates that combine the tumor-targeting ability of monoclonal antibodies with cytotoxic agents (6), offering enhanced cytotoxic efficiency. Due to their specificity, ADCs typically exhibit lower systemic toxicity compared to conventional chemotherapeutic agents, thus offering a more favorable safety profile (7). This, in turn, provides additional therapeutic options for NSCLC patients. Despite this, ADCs still faces many challenges. For instance, due to the limited stability of the systemic circulation of ADCs, only about 1-2% of the administered dose eventually reaches the tumor site, while the remaining 98% May deposit in normal tissues or be released prematurely, thereby causing major adverse events (8).

This article summarizes the latest research progress of ADCs in non-small cell lung cancer, with a focus on elaborating their mechanism of action, key components and clinical applications. Based on this, we focused on discussing the optimization of ADC design, the exploration of combination therapies, and the strengthening of safety management, aiming to promote the research and application of ADCs in the immunotherapy of non-small cell lung cancer.

## 2 Structure and mechanism of action of ADCs

### 2.1 Key components of ADC

The concept of ADCs was first presented by Paul Ehrlich almost 100 years before. He described the antibody as a “magic bullet”: drugs that go straight to their intended cell-structural targets (9). An ADC comprises three essential components: an antibody that binds a tumor-associated antigen, a cytotoxic payload and a connecting linker (10).

#### 2.1.1 Antibody

The antibody serves as the fundamental element in the design of ADCs and must exhibit several essential characteristics, particularly as novel anticancer therapeutics (11). Second, optimized biophysical properties are imperative, encompassing prolonged systemic persistence, diminished immunogenic potential, and stringent target selectivity to avoid off-tissue interactions (12). Finally, molecular compatibility with conjugation chemistries is critical, ensuring stable covalent integration of cytotoxic payloads while preserving antibody integrity during biodistribution, thereby maximizing therapeutic efficacy and minimizing systemic toxicity (13). The human immune system produces five principal immunoglobulin isotypes—IgA, IgD, IgE, IgG, and IgM—each distinguished by structural and functional properties that mediate distinct roles in adaptive immunity. Most ADCs are developed based on IgG (especially IgG1 and IgG4) homologous types because of their excellent solubility and higher affinity for Fcγ receptors, which helps pH-dependent cycling avoid degradation and thus show a longer plasma half-life (14). In other words, this can increase the accumulation of drugs in tumor tissues.

#### 2.1.2 Linkers

Linkers in ADCs bridge the antibody and the cytotoxic drug, serving as key factors influencing ADCs stability and payload release profiles. These linkers must exhibit high stability in plasma to prevent non-specific payload release during circulation in the bloodstream (15). Additionally, linkers should be capable of releasing the cytotoxic drug upon internalization into the target cell. Another critical factor in linker design is its hydrophobic nature. When hydrophobic linkers are combined with cytotoxic payloads that share similar hydrophobic characteristics, they often lead to the aggregation of ADCs molecules. Cut-able and non-cut-able linkers are the main types of linkers adopted by most ADCs, and each type of linker plays a different role in the controlled release and stability of cytotoxic payloads (16). The cleable linkers use the environmental differences between the systemic circulation and tumor cells as “switches” to trigger regulation to release cytotoxic drugs, which mainly include chemical cleable linkers (Hydrazone and disulfide bonds) and enzymatic cleable linkers (glucuronic acid bonds and peptide bonds) (16, 17). For chemical cutting streets, Hydrazone is a typical PH-sensitive connector. The ADC connected to it releases cytotoxic payloads under lysosome pH 4.8 and endosome pH 5.5–6.2 conditions when entering the target cancer cells (18). In addition, the disulfide bonding cleavage joints rely on chemically sensitive cleavage joints that are sensitive to the concentration of reducing glutathione (GSH), which release cytotoxic payloads in cancer cells with elevated GSH (16, 19). For enzymatic cleavage junctions, it is usually necessary to consider proteases that are generally elevated in tumor cells but have inhibited activity or are at low concentrations in plasma. For instance, the β -glucuronide linker is another commonly used enzyme-sensitive linker in ADCs, which can be cleaved by β -glucuronidase (typically highly expressed in tumor areas) to release the payload in the cell (20). In addition, cathepsin B is secreted into the extracellular matrix (ECM), thereby promoting tumor infiltration (21), and the linker targeting this enzyme may have the potential to become a novel linker.

For non-cutting linkers, they usually show inertness to chemical and enzymatic environments, which makes them exhibit high safety. They rely on a certain specific reaction mechanism to release cytotoxic payloads. For instance, cytotoxic payloads coupled with antibody amino acids are released through the enzymatic hydrolysis of ADCs antibody components by proteases (22). Consequently, a meticulously engineered linker serves not only to guarantee precise transportation of the cytotoxic agent to designated therapeutic sites but also crucially contributes to sustaining the structural integrity and preserving the functional efficacy of ADCs during systemic circulation. This dual functionality underscores the linker’s indispensable role in optimizing therapeutic outcomes while mitigating premature degradation or inactivation of the complex.

#### 2.1.3 Cytotoxic payloads

Within ADCs, the cytotoxic moiety serves as the principal therapeutic effector responsible for inducing tumor cell death. Empirical investigations demonstrate that merely 1–2% of systemically delivered ADCs successfully localize to malignant tissues (23), highlighting pharmacokinetic limitations that necessitate the utilization of ultrapotent cytotoxic agents. These findings underscore the imperative for payloads exhibiting subnanomolar cytotoxic activity to compensate for suboptimal tumor accumulation while maintaining an acceptable therapeutic index. For an ADCs to be effective, its payload must possess several key properties. Ideally, the compound should be of relatively low molecular weight and exhibit minimal immunogenicity, thereby reducing the likelihood of immune system interference. In addition, the payload must maintain high stability both in the bloodstream and throughout the endosomal/lysosomal pathways, ensuring that it remains intact until it reaches the target cells. Most importantly, the compound must exhibit potent cytotoxic effects, capable of effectively killing cancer cells upon internalization. Achieving this delicate balance between stability, low immunogenicity, and high cytotoxicity is essential for the optimal performance of ADCs in clinical settings (11, 24, 25). At present, widely utilized cytotoxic agents consist of microtubule inhibitors, DNA-damaging compounds, type I topoisomerase inhibitors, among others (26).

### 2.2 Mechanism of action

**Figure 1:**
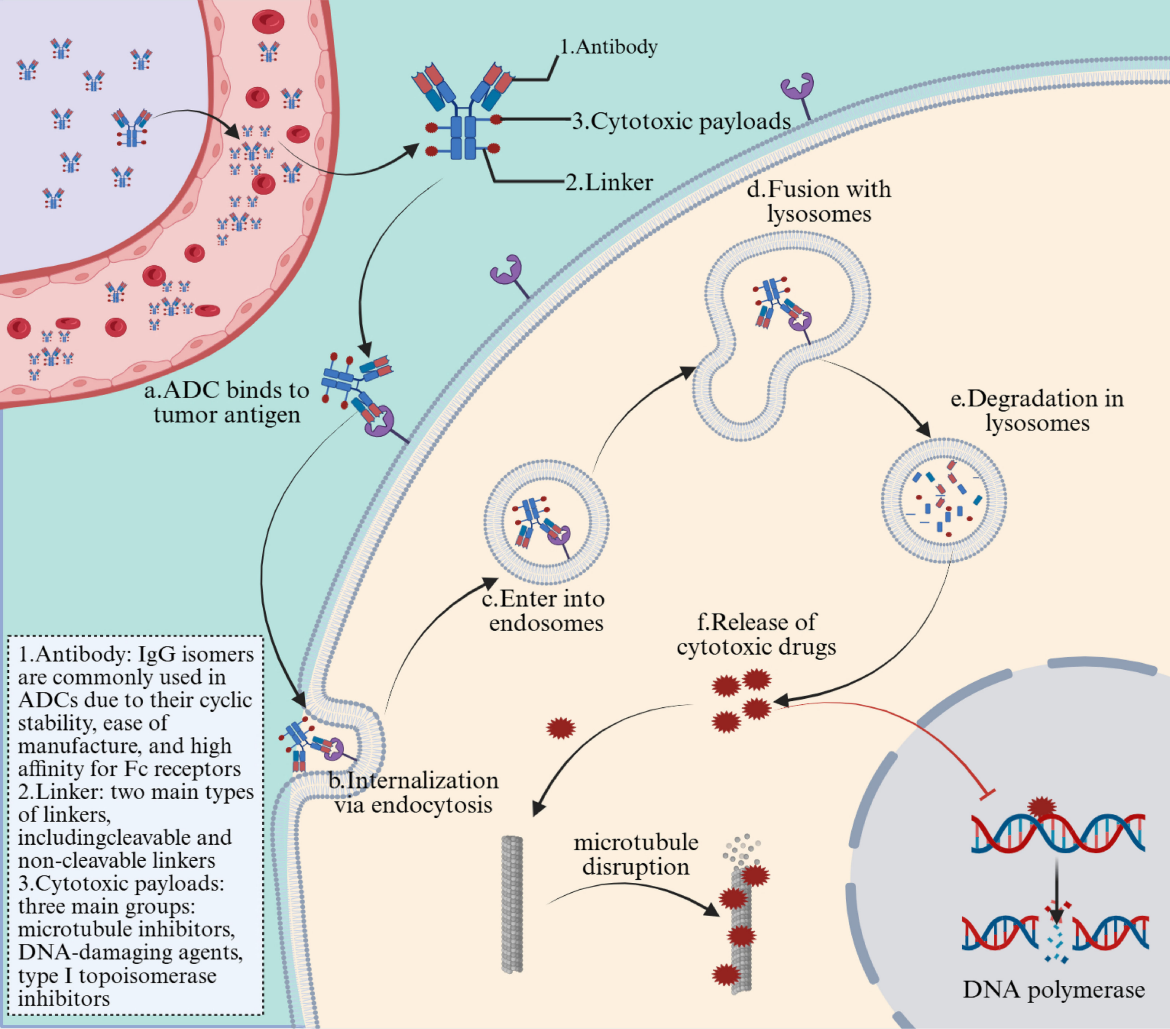
Mechanism of action of antibody-drug conjugates (ADCs) in non-small cell lung cancer (NSCLC).

Upon entering the bloodstream, the antibody components of ADCs recognize and bind to tumor cells that overexpress specific cell surface antigens (27). The ADC-antigen complex is then internalized into the tumor cell via endocytosis (28). Upon internalization, the cytotoxic payload is released through lysosomal degradation. This released payload then interferes with DNA strands or microtubules, or inhibits topoisomerase or RNA polymerase activity, leading to the eventual death or apoptosis of tumor cells (**Figure 1**). If the released payload is capable of permeating cell membranes or is transmembrane in nature, it has the potential to trigger a bystander effect: the cleavable connector can release its payload outside the cell within the tumor tissue and can kill adjacent tumor cells with low or negative expression of the target antigen to enhance the efficacy of ADC (29, 30). Not all ADCs exhibit the bystander effect. These drugs may also influence the tumor microenvironment (TME) through the bystander effect (31).

### 2.3 Advantages and challenges of ADCs

ADCs constitute an emerging category of oncological therapeutics that synergistically combine the specificity of monoclonal antibodies with the potent cytotoxic effects of low-molecular-weight agents. This hybrid architecture facilitates antigen-directed precision, optimized therapeutic payload distribution, and diminished off-target cytotoxicity, collectively enhancing treatment selectivity (32). Despite these advantages, ADCs platforms still face significant challenges, including the emergence of multidrug resistance mechanisms and dose-limiting toxicity related to the drug-antibody ratio (DAR) and payload release.

The occurrence of drug resistance mechanisms is mainly due to the down-regulation of cell surface targets that ADCs rely on, which reduces the effective recognition efficiency of ADCs and subsequently leads to the occurrence of negative event probabilities. Preclinical studies have demonstrated that over time, cells continuously treated with ADCs eventually show a decrease in the expression of target antigen proteins (such as CD30) and other effects (32, 33).

As a key factor in the effective design of ADCs, DAR refers to the average number of drug molecules linked to each antibody (34). The size of DAR can significantly affect the pharmacokinetic characteristics of ADCs, and the degree of drug addition to the antibody can influence the overall stability and aggregation properties of the complex. Although it has been reported that maintaining a DAR at 3–4 usually leads to better pharmacokinetic properties (35). However, a recently developed polymer ADCs shows a DAR value of 20 (36). This emphasizes the need to further explore the optimal DAR values for different ADCs.

As for the toxic events that often occur in ADCs, such as hepatotoxicity, they are often related to their specific payloads. For instance, the calicheamicin payload is associated with an increased incidence of liver injury and hepatotoxicity, although reducing the dose still fails to effectively control the hepatotoxicity it brings (37). These toxic events related to fixed payloads have further stimulated the development of more payloads and demanded more research data and efforts.

## 3 Current status of ADCs in NSCLC treatment

### 3.1 Human epidermal growth factor receptor 2, HER-2

HER2 is an important member of the epidermal growth factor receptor (EGFR) tyrosine kinase family. In NSCLC, the incidences of HER2 overexpression, HER2 amplification and HER2 mutation are 7.7%-23%, 2%-22% and 1%-6.7% respectively, which indicates the application potential of this molecule in the treatment of NSCLC (38). The alteration of HER2 is associated with the poor prognosis of NSCLC, although the guidelines for HER2 testing mainly target breast cancer (38). Unlike other HER family receptors, HER2 does not possess an identified natural ligand. Instead, its downstream signaling pathways are activated via dimerization mechanisms, predominantly through homodimer formation or heterodimer partnerships with HER1 (EGFR) and HER3 (39). HER2 facilitates tumor cell proliferation, cellular survival, metastasis, and invasion in lung cancer via gene mutations, amplification, or aberrant activation of signaling pathways, and is implicated in the development of drug resistance. Some HER2-targeted drugs, such as T-DXd, have shown benefits in patients with advanced NSCLC, indicating that HER2 is not only a prognostic marker but also a promising therapeutic target (38, 40, 41).

#### 3.1.1 Ado-Trastuzumab Emtansine, T-DM1

T-DM1 is the first ADC to be used in the treatment of solid tumors. Trastuzumab is conjugated to the cytotoxic drug DM1 via a non-lysable thioether bond (42). T-DM1 selectively targets and attaches to cells overexpressing HER2 receptors. Following cellular internalization via the HER2-mediated endocytic pathway, the antibody-drug conjugate undergoes lysosomal processing, which cleaves the linker molecule to liberate the DM1 chemotherapeutic agent (43).

In a Phase II biomarker-enriched trial evaluating T-DM1 for HER2-mutant NSCLC harboring exon 20 insertions (n = 22), the therapeutic paradigm demonstrated clinically meaningful signal transduction blockade, evidenced by an objective response rate (ORR) of 38.1% (90% CI: 23.0–55.9%) and a disease control rate (DCR) of 52.4%. Survival kinetics revealed biphasic tumor evolutionary trajectories, with median progression-free survival (mPFS) and median overall survival (mOS) of 2.8 months (90% CI: 1.4–4.3) and 8.1 months (90% CI: 4.5–12.7), respectively, reflecting transient pharmacodynamic durability before adaptive resistance mechanisms emerged.The toxicity de-escalation profile of T-DM1 proved advantageous, with only 14.3% of participants experiencing grade ≥ 3 treatment-emergent adverse events (TEAEs), predominantly hematologic and hepatic perturbations—a finding attributable to T-DM1’s lysosomotropic payload compartmentalization, which minimizes off-target immunogenic sequelae. Notably, the absence of interstitial lung disease or neurotoxicity underscored its targeted cytotoxin delivery mechanism, leveraging HER2-mediated endocytosis for spatial precision (44).

These data position T-DM1 as a molecularly rationalized intervention for HER2-driven NSCLC, particularly in contexts of ligand-independent HER2 dimerization. However, the disconnect between ORR and mPFS suggests transient oncogene dependency, necessitating combinatorial strategies with HER2 degradation inducers (e.g., PROTACs) or immune checkpoint modulators to counteract compensatory pro-survival pathways. Further validation through adaptive basket trials stratifying patients by HER2 extracellular domain conformation and receptor internalization efficiency could refine therapeutic candidacy.

#### 3.1.2 Trastuzumab deruxtecan, T-DXd

As a pioneering biopharmaceutical innovation in precision oncology, T-DXd has achieved global regulatory approval as the first ADC authorized for clinical application in NSCLC. Its structural composition incorporates an engineered human IgG1 monoclonal antibody that selectively binds to HER2 surface receptors, conjugated through a cleavable tetrapeptide-based linker system to a potent topoisomerase I inhibitor therapeutic payload. This configuration optimizes targeted cytotoxicity while maintaining favorable pharmacological profiles through controlled payload release mechanisms (45). The DESTINY-Lung01 trial, a multicenter phase II study employing an open-label, two-cohort design, enrolled 91 HER2-mutated NSCLC patients were administered 6.4 mg/kg T-DXd triweekly. Efficacy evaluations demonstrated an ORR of 55% (95% CI: 44–65%) within the HER2-mutant cohort (n = 91), with a median follow-up duration of 13.1 months. Secondary endpoints included a median duration of response (mDOR) of 9.3 months (95% CI: 5.7–14.7) and mPFS of 8.2 months (95% CI: 6.9–10.8), while mOS reached 17.8 months (95% CI: 13.8–22.1). Safety assessments identified hematological toxicities (e.g., neutropenia, anemia), gastrointestinal disturbances, and treatment-emergent ILD events (26% incidence; two fatalities), underscoring dose-dependent toxicity risks (46). In response to these findings, the subsequent DESTINY-Lung02 trial prospectively randomized 152 HER-2-mutant NSCLC patients (including SNVs and exon 20 insertions) to receive 5.4 mg/kg or 6.4 mg/kg T-DXd regimens. Comparative analysis revealed comparable efficacy between cohorts (mDOR: 12.6 vs. 12.2 months; mPFS: 10.0 vs. 12.9 months; mOS: 19.0 vs. 17.3 months), yet the 5.4 mg/kg cohort exhibited superior tolerability, with reduced rates of treatment discontinuation (14.9% vs. 32.0% for ILD/non-infectious pneumonia) and dose modifications (47). Supported by robust clinical evidence, T-DXd has achieved a Category IIA recommendation in the 2024 CSCO guidelines and stands as the sole preferred second-line therapy for HER2-mutant advanced NSCLC per updated NCCN recommendations. Preliminary data from Cohort 1D of the DESTINY-Lung03 trial, presented at WCLC 2024, evaluated 36 HER2-overexpressing NSCLC patients receiving 5.4 mg/kg T-DXd (median treatment duration: 7.2 months; follow-up: 14.9 months). Outcomes included a confirmed ORR of 44.4%, DCR of 77.8% at 12 weeks, and sustained mDOR of 11.0 months, alongside mPFS and mOS of 8.2 and 17.1 months, respectively. The safety profile aligned with prior studies, reinforcing T-DXd’s therapeutic consistency (48).

### 3.2 Human epidermal growth factor receptor 3, HER3

HER3 is a member of the human epidermal growth factor receptor family. Although its own kinase activity is deficient or negligible, it induces the phosphorylation of receptor tyrosine residues via heterodimerization with other receptor tyrosine kinases (RTKs) (e.g., EGFR and HER2), thereby activating key signaling pathways including PI3K/AKT and MAPK, which drive critical processes in the pathogenesis, progression, and therapeutic resistance of lung cancer (49, 50). Patritumab Deruxtecan (HER3-DXd) epitomizes a third-generation ADC engineered for HER3-tropism, comprising a molecular architecture that integrates a fully human anti-HER3 IgG1 monoclonal antibody (mAb) with Fc-optimized effector function silencing (51). This scaffold is site-specifically conjugated, via a protease-cleavable tetrapeptide linker, to the exatecan-derived topoisomerase I inhibitor payload deruxtecan (DXd)—a membrane-permeable cytotoxin optimized for bystander effect potentiation (52). The clinical effectiveness of HER3-DXd was evaluated in patients with advanced or metastatic NSCLC harboring EGFR mutations and a history of EGFR-TKI therapy in the Phase I dose-expansion study HERTHENA-Lung01. This trial utilized a two-stage dose escalation and expansion cohort design to identify 5.6 mg/kg as the recommended dose for HER3-DXd. Then an additional 45 patients were enrolled, bringing the total number of patients from both the dose escalation and expansion stages to 57, all receiving HER3-DXd at 5.6 mg/kg. The ORR was 39% (95% CI: 26.0% - 52.4%), which exceeded that observed with traditional chemotherapy agents. The mOR was 2.6 months, and the DOR was 6.9 months. Among the 57 patients, 27 (53%) exhibited a reduction in the total tumor diameter of ≥ 30% as measured by imaging following treatment with HER3-DXd. Grade ≥ 3 TEAEs occurred in 64% of patient, with the most common being thrombocytopenia, neutropenia and fatigue (53). Cohort 2 of the dose-expansion phase enrolled 47 previously treated NSCLC patients lacking EGFR-activating mutations, who were not subjected to molecular selection criteria. Therapeutic outcomes included an ORR of 28% and a mPFS duration of 5.4 months (95% confidence interval [CI]: 3.9–12.7) (54).

### 3.3 Trophoblast surface antigen 2, TROP-2

TROP-2 is a transmembrane glycoprotein initially identified in trophoblast cells. TROP-2 is highly expressed in epithelial cancers such as lung adenocarcinoma and is associated with an aggressive oncogenic phenotype (55). Thus, TROP-2 is often considered a potential tumor marker. TROP-2 modulates cell proliferation and survival through the regulation of intracellular calcium concentrations. By interacting with calcium signaling regulatory proteins, TROP-2 plays a pivotal role in governing both the cell cycle and cellular differentiation. Additionally, TROP-2 facilitates cell cycle progression by activating the PI3K/AKT signaling pathway, further contributing to cellular growth and survival mechanisms (56). The intricate biological properties of TROP-2, including its multifaceted downstream signaling pathways and diverse functional roles, have necessitated rigorous safety and efficacy assessments during therapeutic development. Consequently, antibody-drug conjugates targeting TROP-2 remain subjects of active investigation, with the majority currently undergoing preclinical characterization or early-phase clinical evaluation.

#### 3.3.1 Sacituzumab Govitecan (SG, IMMU-132)

IMMU-132 is a humanized monoclonal antibody targeting Trop-2, conjugated through a proprietary hydrolyzable linker to SN-38, the active metabolite of the topoisomerase inhibitor irinotecan (57). IMMU-132 has shown beneficial efficacy for advanced NSCLC (58). In this single-arm multicenter trial, the clinical trial enrolled 54 patients with NSCLC for therapeutic evaluation. Among 47 evaluable subjects (median prior treatment lines: 3; range 2–7), the cohort demonstrated an ORR of 19%, with a mDOR of 6.0 months (95% CI 4.8–8.3). A clinical benefit rate of 43% was observed, defined as complete/partial responses or sustained stable disease (≥4 months). In the intention-to-treat (ITT) population (n = 54), the ORR reached 17% (9/54 cases), with responses emerging at a median onset of 3.8 months, including patients refractory to prior immune checkpoint inhibitors. Survival analysis revealed mPFS of 5.2 months (95% CI 3.2–7.1) and mOS of 9.5 months (95% CI 5.9–16.7). Treatment-related grade ≥ 3 adverse events comprised neutropenia (28%), diarrhea (7%), nausea (7%), fatigue (6%), and febrile neutropenia (4%).

#### 3.3.2 Datopotamab deruxtecan, Dato-DXd

Dato-DXd is an ADC that consists of a humanized IgG1 monoclonal antibody directed against TROP2. This antibody is covalently bound via a protease-cleavable tetrapeptide-based linker, which is specifically engineered to enable controlled release of the cytotoxic payload. The payload is a topoisomerase I inhibitor, designed to exert potent antitumor activity upon delivery to the target site (59). Preliminary data from the TROPI-ON-Pantumor01 Phase I trial—a multicenter dose-escalation/expansion study—revealed clinically meaningful antitumor responses and manageable toxicity parameters in heavily pretreated advanced NSCLC populations (60). TROPION Lung05 is a global Phase II, single-arm, open-label trial aimed at assessing the effectiveness of Dato-DXd in individuals with advanced or metastatic NSCLC who possess specific genomic alterations. The study reported a confirmed ORR of 35.8%, which included four complete responses (CR) and 45 partial response, alongside 56 instances of stable disease. The DCR was 78.8%, with durable responses observed, and the mDOR was 7.0 months. The mPFS was 5.4 months, and the mOS was 13.6 months. In the cohort of 78 patients with EGFR mutations, the confirmed ORR was 43.6%, including all four CRs observed in the study. In the cohort of EGFR-mutated patients, therapeutic outcomes included a mDOR of 7.0 months, an 82.1% DCR, and mPFS and mOS of 5.8 months and 18.3 months, respectively. Within the subgroup of 34 ALK-rearranged cases, the ORR reached 23.5%, with eight individuals achieving partial responses. This group also exhibited a median DOR of 7.0 months, a DCR of 73.5%, and survival metrics of 4.3 months (mPFS) and 9.3 months (mOS). The safety profile was characterized by frequent stomatitis (56%) and nausea (62%), while hematologic adverse drug reactions remained uncommon (61).

#### 3.3.3 Sacituzumab tirumotecan, sac-TMT

Sac-TMT is an innovative ADC targeting TROP-2, which incorporates 2-methylsulfonylpyrimidine as the linker, attached to a topoisomerase I inhibitor (62). In the KL264–01 Phase I/II study, the overall ORR for 43 patients with advanced NSCLC was 40%, while the ORR in the EGFR mutation subgroup (n = 22) reached 55%, significantly higher than the 24% observed in wild-type patients (P = 0.039). Additionally, tumor shrinkage was more pronounced in the EGFR mutation group (-33% vs. -17%, P = 0.036). In the SKB264-II-08 Phase II study, 64 patients with EGFR mutations exhibited an ORR of 34% (95% CI: 23-47) and a DCR of 84% (95% CI: 73-92). Cross-study comparisons demonstrated that patients with EGFR mutations had superior ORRs (KL264-01: 55%; SKB264-II-08: 34%) compared to those receiving conventional chemotherapy (ORR: 3-14%) or other TROP2-targeting ADCs (such as Dato-DXd, which showed a 26% ORR in unscreened populations). Furthermore, the mPFS for patients with EGFR mutations was 11.1 months in KL264-01 (95% CI: 5.7–12.9) and 9.3 months in SKB264-II-08 (95% CI: 7.6–11.4), both longer than the 5.3 months observed in wild-type patients in KL264-01 (P = 0.087). In the cohort of patients with EGFR mutations who were initially treated with chemotherapy (SKB264-II-08 cohort 2), the median PFS was 12.9 months (95% CI: 5.7–21.2). Regarding overall survival (OS), the median OS for patients with EGFR mutations in KL264–01 was 23.0 months (95% CI: 19.7–immature), with a 12-month survival rate of 81% (95% CI: 57–92%). The OS data from SKB264-II-08 are still immature, but the 12-month survival rate has reached 79% (95% CI: 67–87%). The most frequent toxicities were hematologic events, including anemia (84%) and neutropenia (66%). However, serious adverse events, such as interstitial lung disease and diarrhea, were rare, occurring in ≤1% of patients (63). At the same dose, sac-TMT has stronger anti-tumor activity than IMMU-132, and has better safety, and EGFR mutation may be a precise therapeutic target.

### 3.4 MET

The c-MET receptor tyrosine kinase, encoded by the MET proto-oncogene, functions as a important oncogenic signaling nexus among many solid cancers (64). Its activation via hepatocyte growth factor (HGF)-mediated dimerization triggers downstream pathway hyperactivation, including RAS-RAF-MEK-ERK and PI3K-AKT-mTOR cascades, which orchestrate tumor proliferation, metastatic dissemination, and therapeutic resistance (65). NSCLC exhibits a heterogeneous genomic landscape of c-MET dysregulation: overexpression (30–50%), MET exon 14 skipping mutations (3%), and focal amplifications (1.5%)—the latter strongly correlating with EGFR tyrosine kinase inhibitor (TKI) resistance through bypass signaling reactivation. While MET exon 14-targeted TKIs (e.g., capmatinib, tepotinib) have achieved regulatory approval, oncogenic plasticity in MET-overexpressing or amplified tumors remains a therapeutic void, necessitating novel mechanistic polypharmacy approaches.

Telisotuzumab vedotin (Teliso-V) constitutes a pioneering therapeutic entity in targeted oncology, employing a c-MET receptor-targeting antibody-drug conjugate (ADC) architecture. This novel biologic agent is synthesized through precision engineering methodologies, utilizing a site-specific conjugation approach to covalently link the humanized anti-c-MET immunoglobulin G1 (IgG1) monoclonal antibody ABT-700 with the microtubule-inhibitory cytotoxic agent monomethyl auristatin E (MMAE). The conjugation platform employs an enzyme-cleavable valine-citrulline dipeptide linker system, designed to facilitate tumor microenvironment-responsive payload liberation while maintaining circulatory stability. This design achieves controlled drug-to-antibody ratio (DAR) optimization, balancing cytotoxic potency with target-mediated internalization kinetics (66). Early-phase trials revealed heterogeneous clinical translatability: a Phase I dose escalation study demonstrated modest activity in c-MET-overexpressing solid tumors (ORR 18.8%, mPFS 5.7 months), though the SWOG S1400K trial reported limited efficacy (ORR 9%) with grade 5 pulmonary toxicities, prompting early termination (67).

The Phase II LUMINOSITY trial recalibrated Teliso-V’s therapeutic index via predictive biomarker stratification. In 161 c-MET-overexpressing NSCLC patients, tumor-intrinsic MET expression gradients dictated response hierarchies: high- and intermediate-expression cohorts achieved ORRs of 35% and 23%, respectively, with mDOR of 9.0 and 7.2 months, and mOS ≈14 months in both cohorts. The safety profile featured peripheral neuropathy predominance (30%)—a class effect of MMAE—alongside manageable edema (16%) and fatigue (14%), with only two grade 5 TRAEs. These findings underscore Teliso-V’s pharmacodynamic durability in EGFR wild-type, non-squamous NSCLC, irrespective of MET expression thresholds (68).

Current investigations, including the Phase III TeliMET NSCLC-01 and Phase I M14-237 (Teliso-V + osimertinib), aim to resolve adaptive resistance mechanisms such as EMT-driven MET ligand independence or KRAS co-mutations. Challenges persist, however, including pharmacokinetic-pharmacodynamic disjunction in hypovascularized tumors and immune-related adverse events (irAEs) linked to MMAE’s bystander effect. Next-generation strategies propose bispecific MET/EGFR ADCs or immune-stimulatory payloads (e.g., STING agonists) to amplify tumor-selective cytotoxicity while mitigating neurotoxicity.

### 3.5 CEACAM5

CEACAM5, a transmembrane glycoprotein implicated in oncogenic plasticity through its roles in intercellular adhesion and metastatic migration, and is an attractive target for the treatment of ADCs (69). Its overexpression correlates with epithelial- mesenchymal transition (EMT) activation and diminished survival, positioning it as a therapeutic target for precision interception of metastatic dissemination.

Tusamitamab ravtansine, a CEACAM5-directed ADC, exemplifies structural modularity: a humanized monoclonal antibody targeting CEACAM5’s extracellular domain is site-specifically conjugated via a reducible disulfide linker to the maytansinoid DM4 (70), a microtubule-disrupting payload engineered for bystander effect potentiation. Despite demonstrating pharmacokinetic tolerability in Phase I, the Phase III CARMEN LC03 trial failed to meet efficacy endpoints, underscoring the biomarker-refinement imperative in CEACAM5-high populations. However, combinatorial strategies in subsequent studies revealed therapeutic synergy: in the Phase II CARMEN LC04 trial, dual targeting with ramucirumab (anti-VEGFR2) extended mPFS to 5.7 months, while the CARMEN-LC05 study demonstrated immune-chemotherapeutic augmentation, with Tusamitamab ravtansine + pembrolizumab or platinum doublets achieving objective response rates (ORRs) of 47.8% and 59.1%, respectively, and a pooled mPFS of 11.6 months (71, 72).

These divergent outcomes highlight context-dependent efficacy shaped by CEACAM5’s TME adaptability. The ADC’s activity is modulated by CEACAM5 spatial heterogeneity, endocytic recycling rates, and stromal barriers to payload diffusion—factors contributing to pharmacodynamic decoupling in unselected populations. Adverse events, notably peripheral neuropathy (30%) and keratitis, reflect DM4’s axonopathic tropism and corneal epithelial sensitivity to microtubule disruption, necessitating toxicity-mitigated payload engineering in next-generation constructs. The ADCs approved or under development for NSCLC and their major clinical findings are summarized in **Table 1**.

**Table 1:** ADCs approved for NSCLC and major clinical findings.

Target	ADCs	Approved indications	Key clinical research results
HER2	Trastuzumab Deruxtecan (T-DXd)	Advanced NSCLC with HER2 mutation (second-line treatment)	DESTINY-Lung01: ORR 55%, mPFS 8.2 months, mOS 17.8 months; The ORR in the Destiny-Lung 02:5.4 mg/kg dose group was 49%, and the risk of ILD was lower (14.9% vs 32%).
HER2	Trastuzumab Emtansine (T-DM1)	HER2 exon 20 insertion mutation NSCLC (second-line)	Phase II trial: ORR 38.1%, mPFS 2.8 months, mOS 8.1 months; The incidence of grade ≥ 3 TEAEs was 14.3%
TROP2	Sacituzumab Govitecan (SG)	Advanced NSCLC (late-line treatment)	Single-arm trial (n = 54): ORR 17%, mPFS 5.2 months, mOS 9.5 months; Grade ≥ 3 neutropenia is 28%
HER3	Patritumab Deruxtecan (HER3-DXd)	Egfr-tki-resistant EGFR-mutated NSCLC (Under development	Herthena-lung 01: ORR 39%, mPFS 5.4 months; The incidence of grade ≥ 3 TEAEs is 64% (thrombocytopenia is common)
c-MET	Telisotuzumab Vedotin (Teliso-V)	c-MET overexpression NSCLC (Under development)	LUMINOSITY trial: The ORR of the high-expression group was 35%, and the mPFS was 10.0 months. The incidence of peripheral neuropathy is 30%

## 4 Combination of ADCs with immunotherapy

### 4.1 Mechanism of ADCs boosting immune response

One of the key points of this review is to elaborate on the mechanisms of ADCs and immunotherapy and evaluate their combination. ADCs modulate immune activity via a multifaceted mechanism involving the liberation of tumor-associated antigens upon malignant cell lysis, stimulation of dendritic cells (DCs) and other APCs, and subsequent orchestration of downstream immune activation pathways (73). This cascade potentiates adaptive antitumor immunity through coordinated antigen presentation and T-cell priming (74). The core mechanism of ADCs targets specific antigens on tumor cell surfaces, delivering cytotoxic drugs that induce tumor cell death (75). This cell death releases various antigens, including tumor-associated antigens (TAAs) and neoantigens, into the TME. These exposed antigens serve as targets for the immune system, initiating an anti-tumor immune response. Antigen-presenting cells (APCs), such as dendritic cellsand macrophages, play crucial roles in capturing and processing these tumor antigens. DCs internalize tumor antigens via receptors like C-type lectin receptors and present them to T cells as peptide-MHC complexes, which activate a specific immune response. DCs activate cytotoxic T cells (CD8+ T cells) through MHC-I molecules and helper T cells (CD4+ T cells) through MHC-II molecules. Helper T cells assist the immune response by secreting cytokines (e.g., IL-2, IFN-γ) that support CD8+ T cells and B cells (76).

Macrophages also engulf tumor antigens and present them to T cells, further enhancing the immune response (77). Additionally, macrophages regulate the immune microenvironment by secreting cytokines, such as IL-12 and TNF-α, which help amplify the immune response (78, 79). The immunomodulatory sequelae of antibody-drug conjugates (ADCs) transcend direct tumoricidal activity, engendering a dual-phase immunological cascade that orchestrates both immediate neoplastic eradication and durable anti-tumor memory. Mechanistically, ADC-mediated cytotoxicity liberates tumor-associated antigens, which prime clonal expansion of memory T- and B-lymphocyte populations endowed with epigenetic and transcriptional plasticity to mount accelerated anamnestic responses against antigenically related recurrences. This adaptive memory phenotype is further potentiated by ADC-driven immune checkpoint modulation, which circumvents T-cell exhaustion through PD-1/PD-L1 axis disruption.

Concomitantly, ADCs exhibit direct dendritic cell (DC) agonism via structural integration of antibodies targeting co-stimulatory receptors on APCs, such as CD40 or CD11c (80, 81). These immuno-optimized ADC variants function as synthetic DC maturation ligands, inducing upregulation of MHC class II, CD80/86, and CCR7 to enhance migratory competence and T-cell co-stimulation. Furthermore, ADC-induced immunogenic cell death (ICD) releases damage-associated molecular patterns (DAMPs), including HMGB1 and HSPs (82), which act as endogenous adjuvants by binding TLR2/4 and scavenger receptors on DCs. This initiates a paracrine signaling cascade involving type I interferons and IL-12, thereby amplifying cytosolic antigen processing and cross-presentation via MHC-I pathways.

Emerging paradigms leverage spatiotemporal coordination between ADC payloads and immune agonists. For example, protease-activated TLR7/8 ligands conjugated to tumor-targeting antibodies enable tumor site-restricted activation of DCs, minimizing systemic cytokine storms. However, challenges persist, including TME-driven immunosuppression via regulatory T-cell infiltration and adenosine-mediated DC paralysis. To address this, next-generation bispecific ADCs incorporate TGF-β traps or CD39 inhibitors to counteract immunosuppressive circuits while maintaining payload delivery (83). ES014 is a pioneering anti-CD39xTGF-β bispecific antibody (bsAb) that simultaneously targets the adenosine and TGF-β pathways, which are key immunosuppressive mechanisms within the tumor microenvironment (TME). By concurrently blocking CD39 and TGF-β, ES014 inhibits the production of immunosuppressive adenosine, promotes the accumulation of immunostimulatory ATP, and neutralizes the immunosuppressive cytokine TGF-β. Its mechanism involves the targeted delivery of the TGF-β “trap” to CD39-expressing immune cells, thereby blocking adenosine generation through CD39-mediated hydrolysis and reversing TGF-β-induced immunosuppression. This action restores anti-tumor immunity (84). It is worth noting that ES014 can promote the transformation of macrophages in malignant pleural effusion collected from lung cancer patients from the M2 to M1 phenotype, manifested as increased CD86 expression and decreased CD163 expression on CD11b+ cells, which preliminarily indicates its potential in anti-immunosuppression (84). However, as in the ongoing Phase I clinical study, the anti-tumor immune activity of ES014 requires further clinical trial support, despite its good tolerance in cynomolgus monkeys.

### 4.2 Research on the combined application of ADCs and immune checkpoint inhibitors

The combination of immunotherapy and ADC is the current trend in clinical practice, so it is necessary to evaluate their combination and summarize the challenges involved. The combination of ADCs and immune checkpoint inhibitors is currently a promising cancer therapy (85). Immune checkpoint inhibitors, including PD-1/PD-L1 and CTLA-4 antagonists, effectively reverse the immune evasion mechanisms employed by tumor cells, thereby enhancing the body’s immune response against malignancies, thereby enhancing the antitumor activity of T cells. However, their use alone has certain limitations, including limited efficacy in certain patients and immunotolerance in some tumors (86). In these cases, ADCs can play a critical role in enhancing the immune response in two ways: first, by directly killing tumor cells, which exposes additional tumor antigens and strengthens immune recognition; and second, by assisting immune checkpoint inhibitors in overcoming immune escape mechanisms.

When combined with ADCs, immune checkpoint inhibitors can activate previously suppressed T cells, further improving the immune system’s ability to clear tumors. This combination strategy helps overcome tumor immune evasion by amplifying the anti-tumor immune response. The DNA damage fragments caused by ADC can activate the cGAS-STING pathway in tumor cells, produce type I interferon, attract and activate CD8+ T cells and NK cells, transform “cold tumors” into “hot tumors”, and effectively overcome monotherapy resistance of PD-1/PD-L1 (87).

Tumor mutational burden (TMB), which refers to the total number of mutations present within a tumor’s genome, plays a crucial role in determining the immune system’s ability to recognize and attack cancer cells. Generally, tumors with a high TMB are more likely to produce a greater number of tumor-specific antigens, which in turn prompts a stronger immune response (88). Consequently, tumors characterized by high TMB generally exhibit a more favorable response to immune checkpoint inhibitors. Conversely, tumors with low TMB are often associated with a diminished or suboptimal therapeutic response to these treatments (89). In such cases, ADCs can increase TMB by directly targeting tumor cells and inducing cell death, thereby exposing more antigens and further enhancing the immune response, which supports the combined use of immune checkpoint inhibitors.

Thus far, no significant evidence has emerged suggesting that the combination of ICIs with T-DXd (90), Dato-DXd (91), or sacituzumab-govitecan (92) leads to an enhanced risk of toxicity. Notably, the inclusion of ICIs does not seem to exacerbate the occurrence of ILD linked to these ADCs therapies (93). The ongoing Phase III randomized trials (NCT05629585, NCT05382286, and NCT05633654) are positioned to enhance our understanding of this issue. These investigations are expected to shed light on the toxicity profiles and interaction dynamics of combination therapies involving ICIs and ADCs. The current clinical progress of combining various ADCs with ICIs for NSCLC treatment is summarized in **Table 2**.

**Table 2:** Clinical research progress of ADCs combined with ICIs in the treatment of NSCLC.

ADCs	Combined immunotherapy drugs	Key therapeutic outcomes
Trastuzumab Deruxtecan (T-DXd)	Nivolumab (PD-1 Inhibitor)	Her2-positive solid tumors: ORR 63% (significant response in the NSCLC subgroup)
Datopotamab Deruxtecan (Dato-DXd)	Durvalumab (PD-L1 Inhibitor)	a/mTNBC: ORR 77% (NSCLC under expansion)14.3%
Sacituzumab Govitecan (SG)	Pembrolizumab (PD-1 Inhibitor)	Metastatic urothelial carcinoma: ORR 34%
Patritumab Deruxtecan (HER3-DXd)	Osimertinib (EGFR-TKI)	Egfr-mutated NSCLC: Preliminary ORR >50%

## 5 Pharmacokinetics and Pharmacodynamics of ADCs

### 5.1 Absorption, distribution, metabolism and excretion of ADCs

ADCs have a large molecular weight and cannot be effectively absorbed through the gastrointestinal tract; therefore, they are typically administered intravenously (94). Intravenous administration ensures the integrity of ADCs and their rapid distribution throughout the body, via the blood circulatory system, however, a negligible proportion of the administered dose (About 1-2%) of the ADC is effectively delivered to the tumor cells. It also avoids the first-pass effect that may occur when taken orally, ensuring near 100% bioavailability (95). ADCs can release cytotoxic metabolites through two distinct mechanisms: uncoupling and catabolism. Both metabolic pathways typically occur concurrently in the body. The predominant pathway is influenced by various factors, including the stability of the linker, the binding site, and the overall drug load (96).

ADCs typically have a long half-life (ranging from a few days to a week), allowing the drug to remain in the body for an extended period, thereby providing a sustained anti-tumor effect. Due to the structural similarity between ADC and antibodies, the distribution of ADC in the body mirrors that of antibodies and is influenced by many of the same physiological processes. ADC is primarily distributed in the skin, lungs, liver, kidneys, and other tissues (97). The immunoglobulin moiety in ADCs confers precise antigen recognition precision, enabling selective accumulation within malignant tissues through binding to cell membrane-bound tumor-associated antigens. Subsequent intratumoral localization is augmented by the Enhanced Permeability and Retention (EPR) phenomenon (98), a pathophysiological hallmark of neoplastic vasculature. This mechanism exploits the structural aberrations of tumor-associated blood vessels, characterized by hyperpermeable endothelial linings and impaired lymphatic drainage, thereby facilitating enhanced extravasation and prolonged retention of macromolecular therapeutics within the TME (99). However, the size of the EPR effect and the vascular structure of the tumor influence its efficacy across different tumor types. Targeted distribution of the drug can significantly enhance its local concentration in tumor tissue and reduce exposure to normal tissues, thereby minimizing side effects (100).

Systemic ADC concentration levels critically regulate biodistribution patterns, with supraoptimal therapeutic doses potentially inducing antigen saturation at neoplastic sites due to excessive extravascular diffusion. Conversely, subtherapeutic dosing restricts spatial distribution to anatomical regions exhibiting elevated target expression density or heightened vascular permeability—a phenomenon governed by tissue-specific pathophysiological characteristics (101).

ADC is eliminated via metabolic and excretory processes, representing the terminal phase of ADME. The systemic decline in ADC drug concentrations within biological systems predominantly arises from the decomposition of the antibody component, a process mediated by enzymatic activity and supplementary metabolic pathways.

The liver and kidneys are key organs responsible for the metabolism and elimination of drugs. The processes by which drugs are cleared from the body are generally classified into three main categories: metabolic transformation, bile secretion, and renal excretion. Fc receptor-mediated endocytosis is a key mechanism by which the liver clears ADCs. In hepatocytes or related immune cells, ADC is initially endocytosed into endosomes, followed by transfer to lysosomes, where it is degraded into antibody fragments and linker-payload complexes under acidic conditions and protease activity. The released drug payload or its conjugates are further metabolized in liver cells through the oxidative metabolism of cytochrome P450 enzymes (particularly CYP3A4), yielding more polar, easily excreted metabolites (102). After metabolic processing in the liver, the metabolites, now converted into highly water-soluble compounds, enter the intestine primarily via bile and are ultimately excreted in the feces. The molecular weight and polarity of the fully metabolized drug payload, along with certain linker degradation products in the liver, are reduced, these resultant small-molecule metabolites, characterized by their high water solubility, are subsequently filtered through the glomeruli into the urine (103). Some ADCs and their metabolites may also be transported and cleared via the lymphatic system.

### 5.2 Influencing factors

The PK and PD profiles of ADCs are primarily influenced by the characteristics of the antibody, the linker, and the drug payload. A comprehensive understanding of how each of these components contributes to the overall behavior of the ADC is crucial for optimizing its therapeutic efficacy and minimizing potential safety concerns. In the following sections, we delve into the impact of the antibody structure, linker design, and drug loading on both the PK and PD properties of ADCs, exploring the intricate interplay between these factors.

#### 5.2.1 Effect of antibodies on PK and PD of ADCs

The glycosylation of the antibody moiety, the structure of the Fc region, and the mode of conjugation of the antibody to the linker all affect its stability *in vivo* and plasma half-life (104). The most commonly used antibody type is IgG1, it usually has a long half-life (about 2–3 weeks), which helps to prolong the exposure time of the drug in the body and improve the therapeutic effect (105). An antibody’s immunogenicity plays a crucial role in its clearance rate within the body. Increased immunogenicity can lead to early immune-mediated clearance, diminishing the drug’s effectiveness. Additionally, the origin of the antibody (e.g., humanized, chimeric, or murine) and the extent of “humanization” directly influence the likelihood of anti-drug antibody (ADA) formation. The presence of ADAs can alter pharmacokinetic profiles significantly and reduce the therapeutic benefit (106).

The glycosylation patterns and aggregation state of antibodies also impact their recognition as “foreign substances” and their subsequent clearance. Aggregated antibodies are typically cleared more rapidly and are associated with higher immunogenicity. By engineering antibodies to reduce their immunogenicity and aggregation, the *in vivo* longevity of ADCs can be extended, thereby improving the effectiveness of targeted therapy.

#### 5.2.2 Effect of linkers on PK and PD of ADCs

Non-cleavable linkers are known for their exceptional stability in systemic circulation, showing minimal cleavage in plasma. This robust stability minimizes the risk of premature payload release, thereby offering a broader safety margin (107). On the other hand, cleavable linkers, such as hydrazone, disulfide, Val-cit, and β-glucosidase-sensitive linkers (108), can rapidly degrade under the conditions present in the TME or in response to specific enzymes or reducing agents within cells. This degradation facilitates the release of the drug payload, often in its native or nearly native form, which enhances therapeutic efficacy, particularly through the bystander effect (31, 92). Nevertheless, while cleavable linkers offer these advantages, they also pose a higher risk of premature payload release in plasma, potentially leading to increased toxicity.

#### 5.2.3 Effect of cytotoxic payloads on PK and PD of ADCs

Higher DAR values can enhance the cytotoxicity of each ADC molecule by allowing each antibody to carry a greater drug load. However, excessively high DAR values may result in the uncontrolled release of drug loads, leading to nonspecific toxicity to normal cells and potentially reducing the therapeutic efficacy of ADCs (109). Therefore, optimizing the DAR is crucial. Studies have shown that a moderate DAR value (typically between 3 and 6) can balance the anti-tumor effect and safety of the drug, maximizing the therapeutic potential of ADCs (101).

### 5.3 Emerging technologies shaping ADC pharmacokinetics and efficacy

Recent advancements in ADCs have prompted significant innovations aimed at enhancing their PK properties and therapeutic efficacy. The development of novel linkers, payloads, and conjugation techniques has enabled a more precise and targeted delivery of cytotoxic agents to cancer cells, improving the therapeutic index of ADCs.

One of the most promising technological strides is the development of site-specific conjugation. This technique ensures the attachment of cytotoxic payloads to specific sites on the antibody, improving drug stability and minimizing off-target effects. Site-specific conjugation allows for a more controlled and consistent drug-to-antibody ratio (DAR), which directly impacts the pharmacokinetics and efficacy of the ADC. By optimizing the DAR, the conjugate can achieve an ideal balance between tumor-targeting potency and minimizing systemic toxicity (110).

Another key area of innovation is the design of novel payloads. Traditional payloads, such as microtubule inhibitors and DNA-damaging agents, have been effective but come with significant limitations in terms of resistance and off-target toxicity. In response, new classes of payloads, including immune-modulating agents and protease-targeting payloads, are being incorporated into ADCs. These payloads not only enhance the therapeutic efficacy but also aim to overcome common resistance mechanisms, such as alterations in drug metabolism and antigenic loss (111, 112).

Moreover, condition-activatable ADCs are gaining attention as a strategy to further enhance the pharmacokinetics of ADCs. These constructs are designed to remain inert in circulation and are activated only upon reaching the tumor microenvironment, where specific enzymatic or pH changes trigger payload release. This controlled activation minimizes systemic exposure and enhances the targeted delivery of the cytotoxic agent, reducing adverse effects while maximizing tumor kill (113).

These innovations not only improve the precision and potency of ADCs but also provide avenues to overcome resistance and enhance therapeutic outcomes in cancer treatment.

## 6 Resistance mechanisms and strategies in ADCs therapy

The mechanisms underlying drug resistance in ADCs are complex and multifactorial, involving a series of interconnected processes such as alterations in target antigen expression, inactivation of drug payloads, modifications in endocytic pathways, lysosomal dysfunction, disruption of drug release mechanisms, and the influence of the TME. A key resistance mechanism in ADCs is the downregulation of target antigen expression following prolonged exposure to the drug. For instance, reduced HER2 expression was observed in T-eob-DM1-resistant tumor cells, and restoring HER2 expression could reverse the resistance of this cell line to T-eob-DM1 (114). Conversely, the overexpression of target antigens can also limit the effectiveness of ADCs (115).

The internalization of antibodies into tumor cells occurs upon binding to target antigens, facilitated by diverse endocytic pathways. However, subsequent rapid recycling of the antibody-antigen complex can hinder its delivery to lysosomes, where effective drug release requires the transfer of the ADC to this compartment. Upon reaching the lysosome, the cytotoxic agent is typically liberated through chemical or enzymatic cleavage (116). However, resistance may arise when elevated lysosomal pH disrupts the proteolytic environment, inhibiting the cleavage of payloads such as T-DM1 (117).

Another resistance mechanism involves the inability of the cytotoxic agent to effectively traverse from the lysosomal lumen to the cytoplasm, particularly in ADCs with non-cleavable linkers. In these cases, the lysosomal membrane’s impermeability to catabolites necessitates specialized transport mechanisms to shuttle the cytotoxic agents into the cytosol (118). The overexpression of ATP-binding cassette (ABC) transporters in cells leads to the efflux of drugs, thereby reducing the cytotoxic efficacy of anticancer agents. This mechanism is a significant contributor to the development of multidrug resistance (MDR) in human cancers. ABC can use ATP hydrolysis to produce energy to pump the load in the cytoplasm out of tumor cells, leading to tumor multi-resistance to chemotherapy drugs (119). Tumor cells can acquire ADC resistance through the mutation of the target of load (120). In the process of load-induced apoptosis, other related protein mutations can also lead to cell resistance. Currently, addressing the resistance of ADC drugs through combination strategies involving ADCs, chemotherapy drugs, anti-HER2 targeted therapies, and immune checkpoint inhibitors (ICIs), along with the development of new drugs, has become a key focus.

## 7 Safety and toxicity management of ADCs

The following is a summary of the common adverse reactions of ADC drugs and their mechanisms, and the table is attached (**Table 3**).

**Table 3:** Adverse reactions, clinical manifestations, and key mechanisms of drug toxicity.

Adverse reaction	Clinical manifestations	Key mechanisms
Hematologic Toxicity	Neutropenia, anemia, thrombocytopenia	The instability of the linker in circulation leads to the release of “free” cytotoxic drugs, which enter the bone marrow progenitor cells and inhibit proliferation. Some ADC targets are expressed on normal hematopoietic cells, which can directly mediate cellular uptake and cause toxicity.
Gastrointestinal Toxicity	Nausea, vomiting, diarrhea, constipation	Intestinal epithelial cells are sensitive to cytotoxic drugs, and free drugs released in circulation damage the mucosa through passive diffusion or bystander effect.
Interstitial Lung Disease	Dry cough, dyspnea, hypoxia	Macrophage uptake of ADC activates an inflammatory response
Peripheral Neuropathy	Sensory disturbance, muscle weakness	Microtubule inhibitors MMAE interfere with neuronal axon transport, leading to axonal degeneration. Fc receptor-mediated neuronal damage
Hepatic Toxicity	Elevated ALT/AST, liver dysfunction	Some targets are expressed on hepatocytes, causing direct toxicity. Accumulation in the liver after non-specific release of the drug carrier through circulation
Cardiotoxicity	Reduced LVEF, heart failure	Microtubule inhibitors interfere with cardiomyocyte contractile function. The target antigen was expressed in cardiomyocytes.
Infusion-Related Reactions	Fever, chills, hypotension	The Fc region of the antibody binds to the immune cell FcγR and induces mast cell degranulation.
Dermatologic Toxicity	Rash, alopecia, hand-foot syndrome	Payload inhibition of keratinocyte proliferation. Immune complex deposition triggers cytokine release, leading to inflammation

ADC, antibody-drug conjugate; ALT, alanine aminotransferase; AST, aspartate aminotransferase; LVEF, left ventricular ejection fraction; MMAE, monomethyl auristatin E.

## 8 Future perspectives and innovative directions

ADCs is an effective cancer treatment method. The next generation of ADCs will go beyond merely targeting single antigens and delivering cytotoxic agents. The therapeutic landscape of ADCs has diversified to encompass innovative architectures such as bispecific targeting platforms, conditionally activatable constructs, immunomodulatory variants, proteolysis-targeting conjugates, and dual-payload systems (121). Each modality is strategically engineered to circumvent specific limitations in oncology therapeutics, offering tailored mechanisms to address tumor heterogeneity, drug resistance, and microenvironmental complexity.

Precise tumor stratification based on genomic and molecular characteristics has emerged as a key focus in the clinical application of ADCs. The identification of driver mutations (such as EGFR and HER2) and antigen expression profiles (such as TROP2 and Claudin18.2) through comprehensive pan-cancer genomic analysis (e.g., the PCAWG project) can enhance target selection and predict treatment outcomes (122). The effectiveness of Sacituzumab govitecan in EGFR-mutated NSCLC, with an ORR of 55% (63), highlights the potential of mutation status as a biomarker. Research has shown that EGFR mutations can enhance the internalization of antibody-drug conjugates (ADCs) by tumor cells, which in turn improves the efficiency of lysosomal drug delivery. This enhancement leads to a significant increase in drug sensitivity, as evidenced by a 180-fold reduction in the IC50 value (from 15.31 μg/mL to 0.085 μg/mL). Additionally, dynamic monitoring of drug resistance, such as through liquid biopsy, along with the use of combination therapies (e.g., PD-1 inhibitors and PARP inhibitors), offers further potential for improving therapeutic outcomes.

However, the path to personalized ADC therapy is still fraught with challenges. A key hurdle is the comprehensive understanding of drug resistance mechanisms, particularly those that involve the downregulation of target antigens. Another significant issue lies in the complexity of integrating multi-omics data to better inform treatment decisions. Furthermore, the high cost of these therapies remains a barrier to widespread access, particularly for patients in underserved populations.

To address these issues, the development of personalized ADC approaches, using strategies like AI-driven target-payload matching and modular manufacturing technologies (such as WuXiDAR4™), will be pivotal in advancing the field. This shift from traditional “broad-spectrum” cancer treatments to more tailored and precise “targeted elimination” strategies has the potential to revolutionize cancer therapy, ensuring more effective and individualized treatment options for patients.

## 9 Conclusion

In conclusion, ADCs have emerged as a promising therapeutic approach in the treatment of NSCLC, demonstrating substantial progress in both preclinical studies and clinical trials. The combination of monoclonal antibodies, which precisely target tumor cells, with potent cytotoxic agents has significantly improved the specificity and efficacy of cancer therapies, all while minimizing systemic toxicity. ADCs like trastuzumab deruxtecan and sacituzumab govitecan have shown encouraging results, particularly in tumors with specific molecular alterations such as HER2 and TROP2 expression.

Furthermore, the combination of ADCs with ICIs presents an exciting opportunity to overcome immune evasion and enhance overall treatment efficacy, marking an important advancement in cancer immunotherapy. However, several challenges remain, including the development of drug resistance, effective management of toxicity, and the inherent heterogeneity of tumors. Tackling these issues will require continued innovation in ADC design, exploration of more effective combination therapies with immunotherapies, and investigation into novel ADC formats. Personalized medicine, supported by advanced genomic profiling, holds the potential to optimize ADC therapies, allowing for more tailored and effective treatments for NSCLC.

Looking toward the future, the prospects for ADCs in the treatment of NSCLC seem highly promising. Ongoing clinical trials are likely to refine these therapies and could establish them as a cornerstone of NSCLC treatment. The continued evolution of ADC technology, paired with a deeper understanding of resistance mechanisms and better patient selection strategies, will be essential in realizing the full potential of these therapies.

## References

[1] Xia C Dong X Li H Cao M Sun D He S. Cancer statistics in China and United States 2022: profiles, trends, and determinants. Chin Med J. (2022) 135:584–90. doi: 10.1097/CM9.0000000000002108, PMID: 35143424 PMC8920425

[2] Bray F Laversanne M Sung H Ferlay J Siegel RL Soerjomataram I. Global cancer statistics 2022: GLOBOCAN estimates of incidence and mortality worldwide for 36 cancers in 185 countries. CA A Cancer J Clin. (2024) 74:229–63. doi: 10.3322/caac.21834, PMID: 38572751

[3] Fois SS Paliogiannis P Zinellu A Fois AG Cossu A Palmieri G. Molecular epidemiology of the main druggable genetic alterations in non-small cell lung cancer. Int J Mol Sci. (2021) 22:612. doi: 10.3390/ijms22020612, PMID: 33435440 PMC7827915

[4] Howlader N Forjaz G Mooradian MJ Meza R Kong CY Cronin KA. The effect of advances in lung-cancer treatment on population mortality. N Engl J Med. (2020) 383:640–9. doi: 10.1056/NEJMoa1916623, PMID: 32786189 PMC8577315

[5] Mamdani H Matosevic S Khalid AB Durm G Jalal SI. Immunotherapy in lung cancer: current landscape and future directions. Front Immunol. (2022) 13:823618. doi: 10.3389/fimmu.2022.823618, PMID: 35222404 PMC8864096

[6] Chau CH Steeg PS Figg WD. Antibody–drug conjugates for cancer. Lancet. (2019) 394:793–804. doi: 10.1016/S0140-6736(19)31774-X, PMID: 31478503

[7] Ruan D Wu H Meng Q Xu R. Development of antibody-drug conjugates in cancer: Overview and prospects. Cancer Commun. (2024) 44:3–22. doi: 10.1002/cac2.12517, PMID: 38159059 PMC10794012

[8] Tan HN Morcillo MA Lopez J Minchom A Sharp A Paschalis A. Treatment-related adverse events of antibody drug-conjugates in clinical trials. J Hematol Oncol. (2025) 18:71. doi: 10.1186/s13045-025-01720-3, PMID: 40611310 PMC12231679

[9] Strebhardt K Ullrich A. Paul Ehrlich’s magic bullet concept: 100 years of progress. Nat Rev Cancer. (2008) 8:473–80. doi: 10.1038/nrc2394, PMID: 18469827

[10] Hafeez U Parakh S Gan HK Scott AM. Antibody–drug conjugates for cancer therapy. Molecules. (2020) 25:4764. doi: 10.3390/molecules25204764, PMID: 33081383 PMC7587605

[11] Peters C Brown S. Antibody–drug conjugates as novel anti-cancer chemotherapeutics. Bioscience Rep. (2015) 35:e00225. doi: 10.1042/BSR20150089, PMID: 26182432 PMC4613712

[12] Jin Y Schladetsch MA Huang X Balunas MJ Wiemer AJ. Stepping forward in antibody-drug conjugate development. Pharmacol Ther. (2022) 229:107917. doi: 10.1016/j.pharmthera.2021.107917, PMID: 34171334 PMC8702582

[13] Goulet DR Atkins WM. Considerations for the design of antibody-based therapeutics. J Pharm Sci. (2020) 109:74–103. doi: 10.1016/j.xphs.2019.05.031, PMID: 31173761 PMC6891151

[14] Tong JTW Harris PWR Brimble MA Kavianinia I. An insight into FDA approved antibody-drug conjugates for cancer therapy. Molecules. (2021) 26:5847. doi: 10.3390/molecules26195847, PMID: 34641391 PMC8510272

[15] Nagayama A Ellisen LW Chabner B Bardia A. Antibody–drug conjugates for the treatment of solid tumors: clinical experience and latest developments. Targ Oncol. (2017) 12:719–39. doi: 10.1007/s11523-017-0535-0, PMID: 29116596

[16] Fu Z. Antibody drug conjugate: the “biological missile” for targeted cancer therapy. Signal Transduction Targeted Ther. (2022) 7:93. doi: 10.1038/s41392-022-00947-7, PMID: 35318309 PMC8941077

[17] Bargh JD Isidro-Llobet A Parker JS Spring DR. Cleavable linkers in antibody-drug conjugates. Chem Soc Rev. (2019) 48:4361–74. doi: 10.1039/c8cs00676h, PMID: 31294429

[18] Nolting B. Linker technologies for antibody-drug conjugates. Methods Mol Biol. (2013) 1045:71–100. doi: 10.1007/978-1-62703-541-5_5, PMID: 23913142

[19] Zhang D Fourie-O'Donohue A Dragovich PS Pillow TH Sadowsky JD Kozak KR. Catalytic cleavage of disulfide bonds in small molecules and linkers of antibody-drug conjugates. Drug Metab Dispos. (2019) 47:1156–63. doi: 10.1124/dmd.118.086132, PMID: 31085544

[20] Jeffrey SC Nguyen MT Moser RF Meyer DL Miyamoto JB Senter PD. Minor groove binder antibody conjugates employing a water soluble beta-glucuronide linker. Bioorg Med Chem Lett. (2007) 17:2278–80. doi: 10.1016/j.bmcl.2007.01.071, PMID: 17293111

[21] Gondi CS Rao JS. Cathepsin B as a cancer target. Expert Opin Ther Targets. (2013) 17:281–91. doi: 10.1517/14728222.2013.740461, PMID: 23293836 PMC3587140

[22] Erickson HK Widdison WC Mayo MF Whiteman K Audette C Wilhelm SD. Tumor delivery and *in vivo* processing of disulfide-linked and thioether-linked antibody-maytansinoid conjugates. Bioconjug Chem. (2010) 21:84–92. doi: 10.1021/bc900315y.人, PMID: 19891424

[23] Teicher BA Chari RVJ. Antibody conjugate therapeutics: challenges and potential. Clin Cancer Res. (2011) 17:6389–97. doi: 10.1158/1078-0432.CCR-11-1417, PMID: 22003066

[24] Mckertish C Kayser V. Advances and limitations of antibody drug conjugates for cancer. Biomedicines. (2021) 9:872. doi: 10.3390/biomedicines9080872, PMID: 34440076 PMC8389690

[25] Negi A. Antibody–drug conjugates: A comprehensive review. Mol Cancer Res. (2020) 18:3–19. doi: 10.1158/1541-7786.mcr-19-0582, PMID: 31659006

[26] Ponziani S Di Vittorio G Pitari G Cimini AM Ardini M Gentile R. Antibody-drug conjugates: the new frontier of chemotherapy. IJMS. (2020) 21:5510. doi: 10.3390/ijms21155510, PMID: 32752132 PMC7432430

[27] Acchione M Kwon H Jochheim CM Atkins WM. Impact of linker and conjugation chemistry on antigen binding, Fc receptor binding and thermal stability of model antibody-drug conjugates. mAbs. (2012) 4:362–72. doi: 10.4161/mabs.19449, PMID: 22531451 PMC3355488

[28] Jedema I Barge RMY van der Velden VHJ Nijmeijer BA van Dongen JJ Willemze R. Internalization and cell cycle-dependent killing of leukemic cells by Gemtuzumab Ozogamicin: rationale for efficacy in CD33-negative Malignancies with endocytic capacity. Leukemia. (2004) 18:316–25. doi: 10.1038/sj.leu.2403205, PMID: 14614514

[29] Erickson HK Lewis Phillips GD Leipold DD Provenzano CA Mai E Johnson HA. The effect of different linkers on target cell catabolism and pharmacokinetics/pharmacodynamics of trastuzumab maytansinoid conjugates. Mol Cancer Ther. (2012) 11:1133–42. doi: 10.1158/1535-7163.MCT-11-0727, PMID: 22408268

[30] Li F Emmerton KK Jonas M Zhang X Miyamoto JB Setter JR. Intracellular released payload influences potency and bystander-killing effects of antibody-drug conjugates in preclinical models. Cancer Res. (2016) 76:2710–9. doi: 10.1158/0008-5472.CAN-15-1795, PMID: 26921341

[31] Staudacher AH Brown MP. Antibody drug conjugates and bystander killing: is antigen-dependent internalisation required? Br J Cancer. (2017) 117:1736–42. doi: 10.1038/bjc.2017.367, PMID: 29065110 PMC5729478

[32] Drago JZ Modi S Chandarlapaty S. Unlocking the potential of antibody–drug conjugates for cancer therapy. Nat Rev Clin Oncol. (2021) 18:327–44. doi: 10.1038/s41571-021-00470-8, PMID: 33558752 PMC8287784

[33] Chen R Hou J Newman E Kim Y Donohue C Liu X. CD30 downregulation, MMAE resistance, and MDR1 upregulation are all associated with resistance to brentuximab vedotin. Mol Cancer Ther. (2015) 14:1376–84. doi: 10.1158/1535-7163, PMID: 25840583 PMC4458438

[34] Endo Y Shen Y Youssef LA Mohan N Wu WJ. T-DM1-resistant cells gain high invasive activity via EGFR and integrin cooperated pathways. MAbs. (2018) 10:1003–17. doi: 10.1080/19420862.2018.1503904, PMID: 30130447 PMC6260067

[35] Fatima SW Khare SK. Benefits and challenges of antibody drug conjugates as novel form of chemotherapy. J Control Release. (2022) 341:555–65. doi: 10.1016/j.jconrel.2021, PMID: 34906604

[36] Tsuchikama K An Z. Antibody-drug conjugates: recent advances in conjugation and linker chemistries. Protein Cell. (2018) 9:33–46. doi: 10.1007/s13238-016-0323-0, PMID: 27743348 PMC5777969

[37] Dean AQ Luo S Twomey JD Zhang B. Targeting cancer with antibody-drug conjugates: Promises and challenges. MAbs. (2021) 13:1951427. doi: 10.1080/19420862.2021, PMID: 34291723 PMC8300931

[38] Ren S Wang J Ying J Mitsudomi T Lee DH Wang Z. Consensus for HER2 alterations testing in non-small-cell lung cancer. ESMO Open. (2022) 7:100395. doi: 10.1016/j.esmoop.2022.100395, PMID: 35149428 PMC8844658

[39] Del Re M Cucchiara F Petrini I Fogli S Passaro A Crucitta S. erbB in NSCLC as a molecular target: current evidences and future directions. ESMO Open. (2020) 5:e000724. doi: 10.1136/esmoopen-2020-000724, PMID: 32820012 PMC7443272

[40] Arcila ME Chaft JE Nafa K Roy-Chowdhuri S Lau C Zaidinski M. Prevalence, clinicopathologic associations, and molecular spectrum of *ERBB2* ( *HER2* ) tyrosine kinase mutations in lung adenocarcinomas. Clin Cancer Res. (2012) 18:4910–8. doi: 10.1158/1078-0432.CCR-12-0912, PMID: 22761469 PMC3865806

[41] Kris MG Johnson BE Berry LD Kwiatkowski DJ Iafrate AJ Wistuba II. Using multiplexed assays of oncogenic drivers in lung cancers to select targeted drugs. Jama-journal of The American Medical Association (2014) 311:1998–2006. doi: 10.1001/jama.2014.3741, PMID: 24846037 PMC4163053

[42] Verma S Miles D Gianni L Krop IE Welslau M Baselga J. Trastuzumab emtansine for HER2-positive advanced breast cancer. N Engl J Med. (2012) 367:1783–91. doi: 10.1056/nejmoa1209124, PMID: 23020162 PMC5125250

[43] Wolska-Washer A. Safety and tolerability of antibody-drug conjugates in cancer. Drug Safety. (2019) 42:295–314. doi: 10.1007/s40264-018-0775-7, PMID: 30649747 PMC6399172

[44] Iwama E Zenke Y Sugawara S Daga H Morise M Yanagitani N. Trastuzumab emtansine for patients with non–small cell lung cancer positive for human epidermal growth factor receptor 2 exon-20 insertion mutations. Eur J Cancer. (2022) 162:99–106. doi: 10.1016/j.ejca.2021.11.021, PMID: 34959152

[45] Indini A Rijavec E Grossi F. Trastuzumab deruxtecan: changing the destiny of HER2 expressing solid tumors. IJMS. (2021) 22:4774. doi: 10.3390/ijms22094774, PMID: 33946310 PMC8125530

[46] Li BT Smit EF Goto Y Nakagawa K Udagawa H Mazières J. Trastuzumab deruxtecan in *HER2* -mutant non–small-cell lung cancer. N Engl J Med. (2022) 386:241–51. doi: 10.1056/NEJMoa2112431, PMID: 34534430 PMC9066448

[47] Goto K Goto Y Kubo T Ninomiya K Kim SW Planchard D. Trastuzumab deruxtecan in patients with *HER2* -mutant metastatic non–small-cell lung cancer: primary results from the randomized, phase II DESTINY-lung02 trial. JCO. (2023) 41:4852–63. doi: 10.1200/JCO.23.01361, PMID: 37694347 PMC10617843

[48] Planchard D Kim HR Suksombooncharoen T. Trastuzumab deruxtecan monotherapy in pretreated HER2-overexpressing nonsquamous non-small cell lung cancer: DESTINY-lung03 part. doi: 10.1016/j.jtho.2024.09.082

[49] Gandullo-Sánchez L Ocaña A Pandiella A. HER3 in cancer: from the bench to the bedside. J Exp Clin Cancer Res. (2022) 41:310. doi: 10.1186/s13046-022-02515-x, PMID: 36271429 PMC9585794

[50] Scharpenseel H Hanssen A Loges S Mohme M Bernreuther C Peine S. EGFR and HER3 expression in circulating tumor cells and tumor tissue from non-small cell lung cancer patients. Sci Rep. (2019) 9:7406. doi: 10.1038/s41598-019-43678-6, PMID: 31092882 PMC6520391

[51] Yonesaka K Takegawa N Watanabe S Haratani K Kawakami H Sakai K. An HER3-targeting antibody–drug conjugate incorporating a DNA topoisomerase I inhibitor U3–1402 conquers EGFR tyrosine kinase inhibitor-resistant NSCLC. Oncogene. (2019) 38:1398–409. doi: 10.1038/s41388-018-0517-4, PMID: 30302022

[52] Yonesaka K Tanizaki J Maenishi O Haratani K Kawakami H Tanaka K. HER3 augmentation via blockade of EGFR/AKT signaling enhances anticancer activity of HER3-targeting patritumab deruxtecan in EGFR-mutated non–small cell lung cancer. Clin Cancer Res. (2022) 28:390–403. doi: 10.1158/1078-0432.CCR-21-3359, PMID: 34921025

[53] Jänne PA Baik C Su WC Johnson ML Hayashi H Nishio M. Efficacy and safety of patritumab deruxtecan (HER3-DXd) in EGFR inhibitor– resistant, EGFR-mutated non–small cell lung cancer. Cancer Discovery. (2022) 12:74–89. doi: 10.1158/2159-8290.CD-22-0365, PMID: 34548309 PMC9401524

[54] Steuer CE Hayashi H Su WC Nishio M Johnson ML Kim DW. Efficacy and safety of patritumab deruxtecan (HER3-DXd) in advanced/metastatic non-small cell lung cancer (NSCLC) without. EGFR -activating mutations JCO. (2022) 40:9017–7. doi: 10.1200/JCO.2022.40.16_suppl.9017

[55] Santin AD Corr BR Spira A Willmott L Butrynski J Tse KY. Efficacy and safety of sacituzumab govitecan in patients with advanced solid tumors (TROPiCS-03): analysis in patients with advanced endometrial cancer. J Clin Oncol. (2024) 42:3421–9. doi: 10.1200/JCO.23.02767, PMID: 39083724 PMC11458108

[56] Cai J Xu L Tang H Yang Q Yi X Fang Y. The role of the PTEN/PI3K/akt pathway on prognosis in epithelial ovarian cancer: A meta-analysis. Oncologist. (2014) 19:528–35. doi: 10.1634/theoncologist.2013-0333, PMID: 24718516 PMC4012960

[57] Bardia A Mayer IA Vahdat LT Tolaney SM Isakoff SJ Diamond JR. Sacituzumab govitecan-hziy in refractory metastatic triple-negative breast cancer. N Engl J Med. (2019) 380:741–51. doi: 10.1056/NEJMoa1814213, PMID: 30786188

[58] Heist RS Guarino MJ Masters G. Therapy of advanced non–small-cell lung cancer with an SN-38-anti-trop-2 drug conjugate, sacituzumab govitecan. JCO. (2017) 35:2790–7. doi: 10.1200/JCO.2016.72.1894, PMID: 28548889

[59] Dent RA Cescon DW Bachelot T Jung KH Shao ZM Saji S. TROPION-Breast02: Datopotamab deruxtecan for locally recurrent inoperable or metastatic triple-negative breast cancer. Future Oncol. (2023) 19:2349–59. doi: 10.2217/fon-2023-0228, PMID: 37526149

[60] Shimizu T Sands J Yoh K Spira A Garon EB Kitazono S. First-in-human, phase I dose-escalation and dose-expansion study of trophoblast cell-surface antigen 2–directed antibody-drug conjugate datopotamab deruxtecan in non–small-cell lung cancer: TROPION-panTumor01. JCO. (2023) 41:4678–87. doi: 10.1200/JCO.23.00059, PMID: 37327461 PMC10564307

[61] Sands J Ahn MJ Lisberg A Cho BC Blumenschein G Shum E. Datopotamab deruxtecan in advanced or metastatic non–small cell lung cancer with actionable genomic alterations: results from the phase II TROPION-lung05 study. JCO. (2025) 43:1254–65. doi: 10.1200/JCO-24-01349, PMID: 39761483 PMC11949215

[62] Li M Jin M Peng H Wang H Shen Q Zhang L. Current status and future prospects of TROP-2 ADCs in lung cancer treatment. DDDT. (2024) 18:5005–21. doi: 10.2147/DDDT.S489234, PMID: 39525044 PMC11550919

[63] Zhao S Cheng Y Wang Q Li X Liao J Rodon J. Sacituzumab tirumotecan in advanced non-small-cell lung cancer with or without EGFR mutations: phase 1/2 and phase 2 trials. Nat Med. (2025) 31:1976–86. doi: 10.1038/s41591-025-03638-2, PMID: 40210967

[64] Kron A Scheffler M Heydt C Ruge L Schaepers C Eisert AK. Genetic heterogeneity of MET-aberrant NSCLC and its impact on the outcome of immunotherapy. J Thorac Oncol. (2021) 16:572–82. doi: 10.1016/j.jtho.2020.11.017, PMID: 33309988

[65] Uliano J Corvaja C Curigliano G Tarantino P. Targeting HER3 for cancer treatment: a new horizon for an old target. ESMO Open. (2023) 8:100790. doi: 10.1016/j.esmoop.2023.100790, PMID: 36764093 PMC9929675

[66] Wang J Anderson MG Oleksijew A Vaidya KS Boghaert ER Tucker L. ABBV-399, a c-Met Antibody–Drug Conjugate that Targets Both *MET* –Amplified and c-Met–Overexpressing Tumors, Irrespective of *MET* Pathway Dependence. Clin Cancer Res. (2017) 23:992–1000. doi: 10.1158/1078-0432.CCR-16-1568, PMID: 27573171

[67] Strickler JH Weekes CD Nemunaitis J Ramanathan RK Heist RS Morgensztern D. First-in-Human Phase I, Dose-escalation and -expansion study of telisotuzumab vedotin, an antibody–drug conjugate targeting c-met, in patients with advanced solid tumors. JCO. (2018) 36:3298–306. doi: 10.1200/JCO.2018.78.7697, PMID: 30285518

[68] Camidge DR Bar J Horinouchi H Goldman J Moiseenko F Filippova E. Telisotuzumab vedotin monotherapy in patients with previously treated c-met protein–overexpressing advanced nonsquamous *EGFR* -wildtype non–small cell lung cancer in the phase II LUMINOSITY trial. JCO. (2024) 42:3000–11. doi: 10.1200/JCO.24.00720, PMID: 38843488 PMC11361350

[69] Kim YJ Li W Zhelev DV Mellors JW Dimitrov DS Baek DS. Chimeric antigen receptor-T cells are effective against CEACAM5 expressing non-small cell lung cancer cells resistant to antibody-drug conjugates. Front Oncol. (2023) 13:1124039. doi: 10.3389/fonc.2023.1124039, PMID: 36923424 PMC10010383

[70] Pouzin C Tod M Chadjaa M Fagniez N Nguyen L. Covariate analysis of tusamitamab ravtansine, a DM4 anti-CEACAM5 antibody-drug conjugate, based on first-in-human study. CPT Pharmacom Syst Pharma. (2022) 11:384–94. doi: 10.1002/psp4.12769, PMID: 35191618 PMC8923727

[71] Rodriguez Abreu D Veillon R Ravoire M. 1311P Phase II, open-label study of frontline tusamitamab ravtansine with pembrolizumab ± chemotherapy in advanced non-squamous non-small cell lung cancer: Updated results from CARMEN-LC05 trial. Ann Oncol. (2024) 35:S834. doi: 10.1016/j.annonc.2024.08.1368

[72] Dy GK Cho BC Oliveira J. 1411P Tusamitamab ravtansine plus ramucirumab as 2L therapy or beyond in patients with metastatic NSq NSCLC and high CEACAM5 expression (CARMEN-LC04). Ann Oncol. (2023) 34:S807. doi: 10.1016/j.annonc.2023.09.2443

[73] Lv Y Cui X Li T Liu C Wang A Wang T. Mechanism of action and future perspectives of ADCs in combination with immune checkpoint inhibitors for solid tumors. Clin Exp Med. (2025) 25:139. doi: 10.1007/s10238-025-01655-6, PMID: 40319436 PMC12050234

[74] Nicolò E Giugliano F Ascione L Tarantino P Corti C Tolaney SM. Combining antibody-drug conjugates with immunotherapy in solid tumors: current landscape and future perspectives. Cancer Treat Rev. (2022) 106:102395. doi: 10.1016/j.ctrv.2022.102395, PMID: 35468539

[75] Marei HE Cenciarelli C Hasan A. Potential of antibody–drug conjugates (ADCs) for cancer therapy. Cancer Cell Int. (2022) 22:255. doi: 10.1186/s12935-022-02679-8, PMID: 35964048 PMC9375290

[76] Wculek SK. Dendritic cells in cancer immunology and immunotherapy. Nature Reviews Immunology. (2020) 20:7–24. doi: 10.1038/s41577-019-0210-z, PMID: 31467405

[77] Christofides A Strauss L Yeo A Cao C Charest A Boussiotis VA. The complex role of tumor-infiltrating macrophages. Nat Immunol. (2022) 23:1148–56. doi: 10.1038/s41590-022-01267-2, PMID: 35879449 PMC10754321

[78] Wang J Mi S Ding M Li X Yuan S. Metabolism and polarization regulation of macrophages in the tumor microenvironment. Cancer Letters. (2022) 543:215766. doi: 10.1016/j.canlet.2022.215766, PMID: 35690285

[79] Wang H Yung MMH Ngan HYS Chan KKL Chan DW. The impact of the tumor microenvironment on macrophage polarization in cancer metastatic progression. IJMS. (2021) 22:6560. doi: 10.3390/ijms22126560, PMID: 34207286 PMC8235734

[80] Zhou Y Richmond A Yan C. Harnessing the potential of CD40 agonism in cancer therapy. Cytokine Growth Factor Rev. (2024) 75:40–56. doi: 10.1016/j.cytogfr.2023.11.002, PMID: 38102001 PMC10922420

[81] Sum E Rapp M Dürr H Mazumdar A Romero PJ Trumpfheller C. The tumor-targeted CD40 agonist CEA-CD40 promotes T cell priming via a dual mode of action by increasing antigen delivery to dendritic cells and enhancing their activation. J Immunother Cancer. (2022) 10:e003264. doi: 10.1136/jitc-2021-003264, PMID: 35292514 PMC8928381

[82] Wang L Guan R Xie L Liao X Xiong K Rees TW. An ER-targeting iridium(III) complex that induces immunogenic cell death in non-small-cell lung cancer. Angew Chem Int Ed Engl. (2021) 60:4657–65. doi: 10.1002/anie.202013987, PMID: 33217194

[83] Pitt JM Kroemer G Zitvogel L. Immunogenic and Non-immunogenic Cell Death in the Tumor Microenvironment. In: Kalinski P, editor. Tumor Immune Microenvironment in Cancer Progression and Cancer Therapy, vol. 1036. Springer International Publishing (2017). p. 65–79. doi: 10.1007/978-3-319-67577-0_5, PMID: 29275465

[84] Sun D Zhu Y Sun J Meng Z Qiu Q Qiu Y. Preclinical characterization of a novel anti-CD39/TGFβ-trap bispecific antibody that aims to modulate tumor microenvironment. JCO. (2023) 41:e14523–3. doi: 10.1200/JCO.2023.41.16_suppl.e14523

[85] Yu P Zhu C You X Gu W Wang X Wang Y. The combination of immune checkpoint inhibitors and antibody-drug conjugates in the treatment of urogenital tumors: a review insights from phase 2 and 3 studies. Cell Death Dis. (2024) 15:433. doi: 10.1038/s41419-024-06837-w, PMID: 38898003 PMC11186852

[86] Naimi A Mohammed RN Raji A Chupradit S Yumashev AV Suksatan W. Tumor immunotherapies by immune checkpoint inhibitors (ICIs); the pros and cons. Cell Commun Signal. (2022) 20:44. doi: 10.1186/s12964-022-00854-y, PMID: 35392976 PMC8991803

[87] Huang M Cha Z Liu R Lin M Gafoor NA Kong T. Enhancing immunotherapy outcomes by targeted remodeling of the tumor microenvironment via combined cGAS-STING pathway strategies. Front Immunol. (2024) 15:1399926. doi: 10.3389/fimmu.2024.1399926, PMID: 38817608 PMC11137211

[88] Wang X Lamberti G Di Federico A Alessi J Ferrara R Sholl ML. Tumor mutational burden for the prediction of PD-(L)1 blockade efficacy in cancer: challenges and opportunities. Ann Oncol. (2024) 35:508–22. doi: 10.1016/j.annonc.2024.03.007, PMID: 38537779

[89] Jardim DL Goodman A De Melo Gagliato D Kurzrock R. The challenges of tumor mutational burden as an immunotherapy biomarker. Cancer Cell. (2021) 39:154–73. doi: 10.1016/j.ccell.2020.10.001, PMID: 33125859 PMC7878292

[90] Hamilton E Galsky MD Ochsenreither S Del Conte G Martín M De Miguel MJ. Trastuzumab deruxtecan with nivolumab in HER2-expressing metastatic breast or urothelial cancer: analysis of the phase ib DS8201-A-U105 study. Clin Cancer Res. (2024) 30:5548–58. doi: 10.1158/1078-0432.CCR-24-1513, PMID: 39405343 PMC11647201

[91] Schmid P Jung KH Wysocki PJ. 166MO Datopotamab deruxtecan (Dato-DXd) + durvalumab (D) as first-line (1L) treatment for unresectable locally advanced/metastatic triple-negative breast cancer (a/mTNBC): Initial results from BEGONIA, a phase Ib/II study. Ann Oncol. (2022) 33:S199. doi: 10.1016/j.annonc.2022.03.185 42167437

[92] Grivas P Pouessel D Park CH Barthelemy P Bupathi M Petrylak DP. Sacituzumab govitecan in combination with pembrolizumab for patients with metastatic urothelial cancer that progressed after platinum-based chemotherapy: TROPHY-U-01 cohort 3. Journal of Clinical Oncology. (2024) 42:1415–25. doi: 10.1200/JCO.22.02835, PMID: 38261969 PMC11095901

[93] Kroemer G Galluzzi L Kepp O Zitvogel L. Immunogenic cell death in cancer therapy. Annu Rev Immunol. (2013) 31:51–72. doi: 10.1146/annurev-immunol-032712-100008, PMID: 23157435

[94] Deslandes A. Comparative clinical pharmacokinetics of antibody-drug conjugates in first-in-human Phase 1 studies. mAbs. (2014) 6:859–70. doi: 10.4161/mabs.28965, PMID: 24852950 PMC4171021

[95] Chang HP Le HK Shah DK. Pharmacokinetics and pharmacodynamics of antibody-drug conjugates administered via subcutaneous and intratumoral routes. Pharmaceutics. (2023) 15:1132. doi: 10.3390/pharmaceutics15041132, PMID: 37111619 PMC10142912

[96] Malik P Phipps C Edginton A Blay J. Pharmacokinetic considerations for antibody-drug conjugates against cancer. Pharm Res. (2017) 34:2579–95. doi: 10.1007/s11095-017-2259-3, PMID: 28924691

[97] Yip V Palma E Tesar DB Mundo EE Bumbaca D Torres EK. Quantitative cumulative biodistribution of antibodies in mice: Effect of modulating binding affinity to the neonatal Fc receptor. mAbs. (2014) 6:689–96. doi: 10.4161/mabs.28254, PMID: 24572100 PMC4011913

[98] Wu J. The enhanced permeability and retention (EPR) effect: the significance of the concept and methods to enhance its application. JPM. (2021) 11:771. doi: 10.3390/jpm11080771, PMID: 34442415 PMC8402171

[99] Jasim A Abdelghany S Greish K. Current Update on the Role of Enhanced Permeability and Retention Effect in Cancer Nanomedicine. In: Nanotechnology-Based Approaches for Targeting and Delivery of Drugs and Genes. Elsevier (2017). p. 62–109. doi: 10.1016/B978-0-12-809717-5.00002-6

[100] Khera E. Pharmacokinetic and immunological considerations for expanding the therapeutic window of next-generation antibody–drug conjugates. Biodrugs. (2018) 32:465–80. doi: 10.1007/s40259-018-0302-5, PMID: 30132210

[101] Sun X Ponte JF Yoder NC Laleau R Coccia J Lanieri L. Effects of drug–antibody ratio on pharmacokinetics, biodistribution, efficacy, and tolerability of antibody–maytansinoid conjugates. Bioconjugate Chem. (2017) 28:1371–81. doi: 10.1021/acs.bioconjchem.7b00062, PMID: 28388844

[102] Mahmood I. Clinical pharmacology of antibody-drug conjugates. Antibodies. (2021) 10:20. doi: 10.3390/antib10020020, PMID: 34063812 PMC8161445

[103] Mahmood I. Effect of intrinsic and extrinsic factors on the pharmacokinetics of antibody–drug conjugates (ADCs). Antibodies. (2021) 10:40. doi: 10.3390/antib10040040, PMID: 34698086 PMC8544203

[104] Tarcsa E. Current approaches for ADME characterization of antibody-drug conjugates: An industry white paper. Drug Metab Pharmacokinetics. (2017) 32:S5. doi: 10.1016/j.dmpk.2016.10.026 26669328

[105] Hoffmann RM Coumbe BGT Josephs DH Mele S Ilieva KM Cheung A. Antibody structure and engineering considerations for the design and function of Antibody Drug Conjugates (ADCs). OncoImmunology. (2018) 7:e1395127. doi: 10.1080/2162402X.2017.1395127, PMID: 29375935 PMC5769674

[106] Vaisman-Mentesh A Gutierrez-Gonzalez M DeKosky BJ Wine Y. The molecular mechanisms that underlie the immune biology of anti-drug antibody formation following treatment with monoclonal antibodies. Front Immunol. (2020) 11:1951. doi: 10.3389/fimmu.2020.01951, PMID: 33013848 PMC7461797

[107] Oflazoglu E Stone IJ Gordon K Wood CG Repasky EA Grewal IS. Potent Anticarcinoma Activity of the Humanized Anti-CD70 Antibody h1F6 Conjugated to the Tubulin Inhibitor Auristatin via an Uncleavable Linker. Clin Cancer Res. (2008) 14:6171–80. doi: 10.1158/1078-0432.CCR-08-0916, PMID: 18809969

[108] Balamkundu S Liu CF. Lysosomal-cleavable peptide linkers in antibody–drug conjugates. Biomedicines. (2023) 11:3080. doi: 10.3390/biomedicines11113080, PMID: 38002080 PMC10669454

[109] Perez HL Cardarelli PM Deshpande S Gangwar S Schroeder GM Vite GD. Antibody–drug conjugates: current status and future directions. Drug Discov Today. (2014) 19:869–81. doi: 10.1016/j.drudis.2013.11.004, PMID: 24239727

[110] Hsu YP Nourzaie O Tocher AE Nerella K Ermakov G Jung J. Site-specific antibody conjugation using modified bisected *N*-glycans: method development and potential toward tunable effector function. Bioconjugate Chem. (2023) 34:1633–44. doi: 10.1021/acs.bioconjchem.3c00302, PMID: 37620302 PMC10516122

[111] Wang Z Li H Gou L Li W Wang Y. Antibody–drug conjugates: Recent advances in payloads. Acta Pharm Sin B. (2023) 13:4025–59. doi: 10.1016/j.apsb.2023.06.015, PMID: 37799390 PMC10547921

[112] Wang Y Wang Z Lu Y Shi K Zhang J Wu C Novel payloads of antibody-drug conjugates. In: Drug Discovery Stories. Elsevier. p. 253–68. doi: 10.1016/b978-0-443-23932-8.00014-5

[113] Chang HW Frey G Wang J Liu H Xing C Chen J. Preclinical development of ozuriftamab vedotin (BA3021), a novel ROR2-specific conditionally active biologic antibody–drug conjugate. mAbs. (2025) 17. doi: 10.1080/19420862.2025.2490078, PMID: 40202784 PMC11988251

[114] Loganzo F Tan X Sung M Jin G Myers JS Melamud E. Tumor cells chronically treated with a trastuzumab–maytansinoid antibody–drug conjugate develop varied resistance mechanisms but respond to alternate treatments. Mol Cancer Ther. (2015) 14:952–63. doi: 10.1158/1535-7163.MCT-14-0862, PMID: 25646013

[115] Van Der Velden VHJ Boeckx N Jedema I te Marvelde JG Hoogeveen PG Boogaerts M. High CD33-antigen loads in peripheral blood limit the efficacy of gemtuzumab ozogamicin (Mylotarg®) treatment in acute myeloid leukemia patients. Leukemia. (2004) 18:983–8. doi: 10.1038/sj.leu.2403350, PMID: 15029214

[116] Karcini A Mercier NR Lazar IM. Proteomic assessment of SKBR3/HER2+ breast cancer cellular response to Lapatinib and investigational Ipatasertib kinase inhibitors. Front Pharmacol. (2024) 15:1413818. doi: 10.3389/fphar.2024.1413818, PMID: 39268460 PMC11391243

[117] Ríos-Luci C García-Alonso S Díaz-Rodríguez E Nadal-Serrano M Arribas J Ocaña A. Resistance to the antibody–drug conjugate T-DM1 is based in a reduction in lysosomal proteolytic activity. Cancer Res. (2017) 77:4639–51. doi: 10.1158/0008-5472.CAN-16-3127, PMID: 28687619

[118] Hamblett KJ Jacob AP Gurgel JL Tometsko ME Rock BM Patel SK. SLC46A3 is required to transport catabolites of noncleavable antibody maytansine conjugates from the lysosome to the cytoplasm. Cancer Res. (2015) 75:5329–40. doi: 10.1158/0008-5472.CAN-15-1610, PMID: 26631267

[119] Di Roio A Hubert M Besson L Bossennec M Rodriguez C Grinberg-Bleyer Y. MDR1-expressing CD4^+^ T cells with Th1.17 features resist to neoadjuvant chemotherapy and are associated with breast cancer clinical response. J Immunother Cancer. (2023) 11:e007733. doi: 10.1136/jitc-2023-007733, PMID: 37940345 PMC10632904

[120] Coates JT Sun S Leshchiner I Thimmiah N Martin EE McLoughlin D. Parallel genomic alterations of antigen and payload targets mediate polyclonal acquired clinical resistance to sacituzumab govitecan in triple-negative breast cancer. Cancer Discovery. (2021) 11:2436–445. doi: 10.1158/2159-8290.CD-21-0702, PMID: 34404686 PMC8495771

[121] Tsuchikama K Anami Y Ha SYY Yamazaki CM. Exploring the next generation of antibody–drug conjugates. Nat Rev Clin Oncol. (2024) 21:203–23. doi: 10.1038/s41571-023-00850-2, PMID: 38191923

[122] Savage SR Yi X Lei JT Wen B Zhao H Liao Y. Pan-cancer proteogenomics expands the landscape of therapeutic targets. Cell. (2024) 187:4389–4407.e15. doi: 10.1016/j.cell.2024.05.039, PMID: 38917788 PMC12010439

